# Cancer and the 10-Year Incidence of Chronic Low Back Pain in 407,314 Adults Followed in General Practices in Germany

**DOI:** 10.3390/jcm13226969

**Published:** 2024-11-19

**Authors:** Karel Kostev, Augustin Latourte, Dong Keon Yon, Josep Maria Haro, Pascal Richette, Johann Beaudreuil, Louis Jacob

**Affiliations:** 1 Epidemiology, IQVIA, 60549 Frankfurt, Germany; karel.kostev@iqvia.com; 2 University Clinic, Phillips-University, 35043 Marburg, Germany; 3 AP-HP, Université Paris Cité, Lariboisière Hospital, Department of Rheumatology, 75010 Paris, France; augustin.latourte@aphp.fr (A.L.); pascal.richette@aphp.fr (P.R.); 4 Université Paris Cité, Inserm U1132, Bioscar, 75010 Paris, France; johann.beaudreuil@aphp.fr; 5 Center for Digital Health, Medical Science Research Institute, Kyung Hee University College of Medicine, Seoul 130-701, Republic of Korea; yonkkang@gmail.com; 6 Department of Regulatory Science, Kyung Hee University, Seoul 130-701, Republic of Korea; 7 Department of Pediatrics, Kyung Hee University Medical Center, Kyung Hee University College of Medicine, Seoul 130-701, Republic of Korea; 8 Research and Development Unit, Parc Sanitari Sant Joan de Déu, CIBERSAM, ISCIII, Dr. Antoni Pujadas, 42, 08830 Sant Boi de Llobregat, Barcelona, Spain; josepmaria.haro@sjd.es; 9 AP-HP, Université Paris Cité, Lariboisière-Fernand Widal Hospital, Department of Physical Medicine and Rehabilitation, 75010 Paris, France; 10 Université Paris Cité, Inserm U1153, Epidemiology of Ageing and Neurodegenerative Diseases (EpiAgeing), 75010 Paris, France

**Keywords:** cancer, chronic low back pain, epidemiology, Germany, retrospective cohort study

## Abstract

**Objective:** There is a scarcity of data on the long-term relationship between cancer and chronic low back pain (CLBP). Therefore, this retrospective cohort study investigated the association between cancer and the 10-year incidence of CLBP in Germany. **Methods:** Data collected in 1293 German general practices between 2005 and 2022 were used for the study. Patients diagnosed with cancer were matched to those without cancer (1:1) using a propensity score based on age, sex, the mean number of consultations per year during the follow-up, index year, and several chronic conditions. The index date was the consultation corresponding to cancer diagnosis in the cancer group and a random visit date in the noncancer group. The analyses included Kaplan–Meier curves with the log-rank test and Cox regression models adjusted for other frequent conditions. **Results:** There were 203,657 adults in the cancer group and 203,657 adults in the noncancer group. The mean (SD) age was 66.2 (14.6) and 66.0 (13.8) years in patients with and without cancer, respectively, with a proportion of women of 51.3–51.8%. Within 10 years of the index date, 16.1% of people with cancer and 18.8% of those without cancer were diagnosed with CLBP (*p*-value < 0.001). The Cox regression analysis corroborated this finding, as there was a negative and significant association between cancer and CLBP (HR = 0.87, 95% CI = 0.86–0.89). **Conclusions:** Cancer was not associated with increased odds of CLBP in the decade following its diagnosis in Germany. Due to limitations inherent to the data, caution should be taken when interpreting the study results.

## 1. Introduction

Chronic low back pain (CLBP) corresponds to pain in the lower back lasting more than three months [1]. In 2020, there were 619 million people with low back pain globally [2], with a chronicization rate between 5% and 10% [3]. People with CLBP are at an increased risk for several chronic physical conditions (e.g., diabetes [4] and stroke [5]), decreased quality of life [6], and at older age, higher mortality compared with the general population [7]. Moreover, CLBP is a risk factor for long-term work incapacity [8] and is associated with substantial direct and indirect costs [9]. Taking these data together, it is of the utmost importance to understand better risk factors for CLBP.

In recent years, there has been a growing interest in the association between cancer and chronic pain [10,11,12,13,14,15,16,17]. For example, a study of 115,091 individuals from the United States of America (USA) revealed that cancer was positively and significantly associated with chronic pain (odds ratio = 1.48, 95% confidence interval = 1.38–1.59) [14]. The relationship between cancer and chronic pain could be explained by the cancer itself and its treatment (e.g., chemotherapy, radiotherapy, and surgery) [18]. Although these studies are of interest, none of them focused on CLBP specifically, and there are no comparative data on the incidence of CLBP in the cancer population versus the general population. Besides, the vast majority of this research was of a cross-sectional nature, and the temporality of the cancer–chronic pain relationship could not be formally assessed [10,11,12,13,14,15,16]. Third, more than half of the studies were conducted in Norway [11,12] or the USA [10,13,14,15], and the generalizability of their findings to other countries may be limited. In light of these limitations, data on the relationship between cancer and CLBP are urgently needed.

Therefore, the aim of this retrospective cohort study was to investigate the association between cancer and the 10-year incidence of CLBP in adults followed in general practices in Germany. The hypothesis was that cancer would be associated with a significant increase in the incidence of CLBP. The cancer–incident CLBP relationship could be mediated by unspecific factors also involved in the occurrence of chronic pain at other anatomical sites (see above) and more specific factors, such as decreased physical activity [19,20], multiple chronic physical conditions (e.g., diabetes [21,22] and obesity [23,24]), and psychological distress [25,26].

## 2. Materials and Methods

### 2.1. Ethics Approval and Consent to Participate

The use of anonymous medical data for research is made possible by German law if specific criteria are met. Under this law, approval from a medical ethics committee is unnecessary for this type of study, which does not contain directly identifiable data. In addition, the study does not require informed consent from patients.

### 2.2. Guidelines

This research was conducted in accordance with the STROBE guidelines for cohort studies (see Supplementary Table S1).

### 2.3. Database

Data from the Disease Analyzer database (IQVIA) were used for this research. The detailed methodology of the database is available in the scientific literature [27]. Briefly, the Disease Analyzer database includes data from private general and specialized practices in Germany. These data correspond to sociodemographic variables, diagnoses, and prescriptions. The diagnosis of conditions relies on the International Classification of Diseases, 10th revision (ICD-10). The documentation of prescribed drugs is based on the Anatomical Classification of Pharmaceutical Products of the European Pharmaceutical Market Research Association (EphMRA). Following anonymization, IQVIA receives the data from the computers of the practices every month. Quality assessment relies on several factors (e.g., completeness of information and linkage between conditions and prescribed drugs). The selection of the participating private practices incorporates multiple criteria (i.e., physician age, practice specialty, size of the community, and federal state). Finally, the Disease Analyzer database is representative of primary care practices in Germany [27].

### 2.4. Study Population

**Figure 1:**
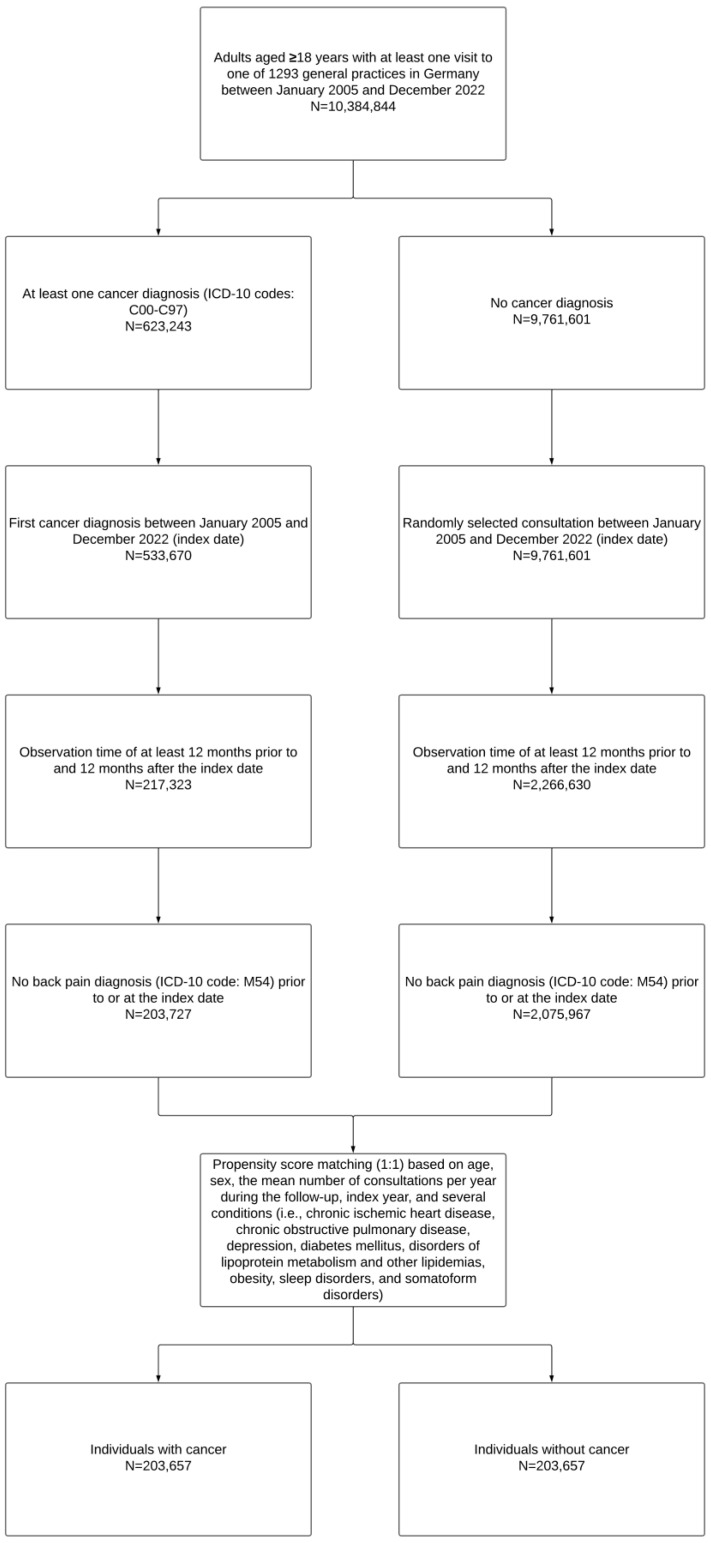
Flow chart of the study participants.

This study focused on adults aged ≥18 years who received a diagnosis of cancer for the first time (ICD-10 codes: C00–C97) in one of 1293 general practices in Germany during the period January 2005–December 2022 (index date). To be included in the analyses, individuals had to be followed in the practice for at least 12 months before the index date and followed for at least 12 months after the index date, as well as no back pain diagnosis (ICD-10 code: M54) within the 12-month period prior to the index date. The same inclusion criteria were applied to identify people without cancer who were matched (1:1) to those with cancer using a propensity score. The variables included in the propensity score were age, sex, the mean number of consultations per year during the follow-up, index year, and several chronic conditions documented within the 12-month period before or at the index date (i.e., chronic ischemic heart disease [ICD-10 code: I25], chronic obstructive pulmonary disease [ICD-10 code: J44], depression [ICD-10 codes: F32 and F33], diabetes mellitus (ICD-10 codes: E10–E14), disorders of lipoprotein metabolism and other lipidemias [ICD-10 code: E78], obesity [ICD-10 code: E66], sleep disorders [ICD-10 code: G47], and somatoform disorders [ICD-10 code: F45]). The above disorders were included in the propensity score, as these conditions are known to be associated with both cancer and low back pain. In adults without cancer, the index date was a random consultation in the period January 2005–December 2022. Patients with and without cancer could have died during the follow-up; although, data on mortality are not available in the Disease Analyzer database. Finally, the flow chart of the study participants is displayed in Figure 1.

### 2.5. Study Outcome

The dependent variable of the study was incident CLBP in adults with and without cancer within 10 years of the index date. Following prior research [21], CLBP corresponded to the presence of two diagnoses of low back pain with or without sciatica (ICD-10 codes: M54.4 [i.e., lumbago with sciatica] and M54.5 [i.e., low back pain]) with more than three months between the diagnoses.

### 2.6. Study Covariates

Covariates were age (continuous and categorical [i.e., ≤50, 51–60, 61–70, 71–80, and >80 years]), sex (i.e., female and male), the mean number of consultations per year during the follow-up, index year (i.e., 2005–2007, 2008–2010, 2011–2013, 2014–2016, 2017–2019, and 2020–2022), chronic conditions included in the propensity score (i.e., chronic ischemic heart disease, chronic obstructive pulmonary disease, depression, diabetes mellitus, disorders of lipoprotein metabolism and other lipidemias, obesity, sleep disorders, and somatoform disorders), and other chronic conditions not included in the propensity score. These later conditions, which were diagnosed in at least 5% of adults with cancer within 12 months prior to or at the index date, were atrial fibrillation and flutter (ICD-10 code: I48), disorders of the thyroid gland (ICD-10 codes: E00–E07), essential hypertension (ICD-10 code: I10), gastritis and duodenitis (ICD-10 code: K29), gastro-esophageal reflux disease (ICD-10 code: K21), heart failure (ICD-10 code: I50), osteoarthritis of the knee (ICD-10 code: M17), and varicose veins of the lower extremities (ICD-10 code: I83). Cancer diagnosis included breast cancer (ICD-10 code: C50), cancer of bronchus and lung (ICD-10 code: C34), cancer of esophagus or stomach (ICD-10 codes: C15 and C16), cancer of female genital organs (ICD-10 codes: C51–C58), colorectal cancer (ICD-10 codes: C18 and C20), malignant neoplasms of ill-defined, other secondary, and unspecified sites (ICD-10 codes: C76–C80), malignant neoplasms of lymphoid, hematopoietic and related tissue (ICD-10 codes: C81–C96), prostate cancer (ICD-10 code: C61), skin cancer (ICD-10 codes: C43 and C44), urinary tract cancer (ICD-10 codes: C64–C68), and cancer of other sites. Finally, the presence of metastasis (ICD-10 codes: C77–C79; these codes were also part of those used for malignant neoplasms of ill-defined, other secondary, and unspecified sites) was documented at the index date and within the first six months of follow-up.

### 2.7. Statistical Analyses

The characteristics of adults with and without cancer were described using the mean (standard deviation) for numerical variables and the absolute number (proportion) for categorical variables. The variables included in the propensity score (i.e., age, sex, the mean number of consultations per year during the follow-up, index year, and several chronic conditions) were compared between the two groups using the standardized mean difference (SMD). This statistic is frequently used to analyze the distribution of covariates after propensity score matching, with differences higher than 0.1 corresponding to some imbalance [28]. Other chronic conditions not included in the propensity score were compared between the cancer and the noncancer group using the McNemac test. In addition, the most frequent cancer diagnoses were descriptively described in the cancer cohort. Moreover, the incidence of CLBP in the 10 years within the index date was analyzed in people with and without cancer using Kaplan–Meier curves, and the two curves were compared with the log-rank test. This analysis was repeated in participants without any diagnosis of back pain in their entire medical history prior to or at the index date. Finally, the association between cancer and incident CLBP was investigated with Cox regression models adjusted for chronic conditions not included in the propensity score in the overall sample and by age, sex, and cancer diagnosis. The Cox regression analysis was also conducted in people who had never been diagnosed with back pain prior to or at the index date and in the overall population after excluding participants with skin cancer, as skin cancer is the most frequent cancer type [29] and is frequently considered (at least basal cell carcinoma) as a less severe cancer compared with other types of cancer [30]. Results of the Cox regression analyses are displayed as hazard ratios (HRs) and 95% confidence intervals (CIs). There were no missing data to handle. A two-sided *p*-value lower than 0.050 was considered statistically significant. All analyses were performed using SAS version 9.4 (SAS Institute, Cary, NC, USA).

## 3. Results

### 3.1. Characteristics of the Population

There were 203,657 adults with and 203,657 adults without cancer included in the study. The characteristics of the population are displayed in Table 1. The mean (standard deviation) age was 66.2 (14.6) and 66.0 (13.8) years in the cancer and noncancer group, respectively (SMD = −0.014). The proportion of women was 51.3% in the first group and 51.8% in the second group (SMD = −0.005). The mean (standard deviation) number of consultations per year during the follow-up was 8.7 (3.8) in participants with and 8.8 (3.8) in those without cancer (SMD = −0.026), while the mean (standard deviation) follow-up was 5.3 (3.1) years in the cancer cohort and 4.7 (2.9) years in the noncancer cohort. In terms of the chronic conditions included in the propensity score, disorders of lipoprotein metabolism and other lipidemias (29.0% in the cancer group and 29.0% in the noncancer group), diabetes mellitus (21.1% and 21.6%), and depression (15.8% and 15.9%) were the most frequent disorders. Regarding the chronic conditions not included in the propensity score, the three commonest diseases were essential hypertension (47.4% in the cancer group and 49.1% in the noncancer group; *p*-value < 0.001), disorders of the thyroid gland (21.7% and 21.3%; *p*-value = 0.001), and gastritis and duodenitis (15.4% and 15.7%; *p*-value = 0.006; Table 2). Finally, the three most frequent cancer diagnoses in the cancer group were skin cancer (22.3%), breast cancer (14.7%), and prostate cancer (11.3%; Table 3). The prevalence of metastasis at the index date or within the first six months of follow-up was 4.5%.

### 3.2. Cancer and Incident CLBP

**Figure 2:**
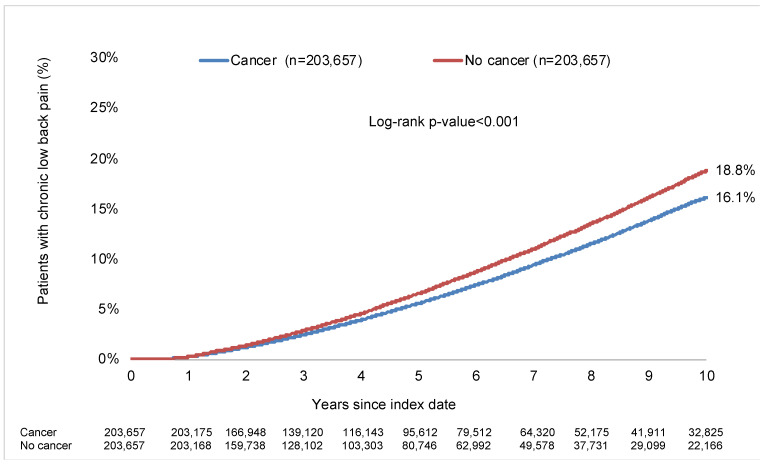
10-year incidence of chronic low back pain in people with and without cancer followed in general practices in Germany. Abbreviation: ICD-10: International Classification of Diseases, 10th revision. Chronic low back pain was defined as the presence of two diagnoses of low back pain with or without sciatica (ICD-10 codes: M54.4 [i.e., lumbago with sciatica] and M54.5 [i.e., low back pain]) with more than three months between the diagnoses.

Kaplan–Meier curves are displayed in Figure 2. Within 10 years of the index date, 16.1% of the cancer group and 18.8% of the noncancer group were diagnosed with CLBP (log-rank *p*-value <0.001). The respective figures in participants never diagnosed with back pain prior to or at the index date were 8.8% and 9.6% (log-rank *p*-value < 0.001; data only shown in the text). After adjusting for comorbidities, cancer was negatively and significantly associated with incident CLBP in the overall population (HR = 0.87, 95% CI = 0.86–0.89; Table 4). This inverse association was corroborated in most stratified analyses, except in people aged >80 years (HR = 1.12, 95% CI = 1.04–1.19). The cancer–CLBP relationship was also not statistically significant in people aged 71–80 years and those with cancer of bronchus and lung, cancer of female genital organs, malignant neoplasms of ill-defined, other secondary, and unspecified sites, and metastasis. Finally, the overall association was also negative and statistically significant in participants with no back pain diagnosis in their entire medical history prior to or at the index date (HR = 0.94, 95% CI = 0.90–0.98), while the relationship was corroborated after excluding skin cancer from the analyses (HR = 0.85, 95% CI = 0.83–0.87; data only shown in the text).

## 4. Discussion

### 4.1. Main Findings

In this retrospective cohort study of more than 407,000 adults followed in general practices in Germany, the 10-year incidence of CLBP was 16.1% in those diagnosed with cancer and 18.8% in those without cancer (log-rank *p*-value < 0.001). After adjusting for frequent comorbidities, the Cox regression analysis further identified a negative and significant association between cancer and incident CLBP in the overall population, with an HR of 0.87. In contrast, there was a positive and significant relationship between cancer and CLBP in adults aged >80 years, while no statistically significant association was observed for those aged 71–80 years and those with specific types of cancer or metastasis. To the best of the authors’ knowledge, this research is the first to investigate the long-term effects of cancer diagnosis on the incidence of CLBP, with no significant increased incident CLBP in cancer survivors.

### 4.2. Interpretation of the Findings

The inverse association between cancer and incident CLBP observed in the study should be interpreted with caution. Before going further, it should be noted that the most frequent cancers in this study were skin, breast, and prostate cancers. However, recent data from the USA have shown that the most common cancers in men are prostate, lung and bronchus, and colorectal cancers, while the respective cancers in women are breast, lung and bronchus, and colorectal cancers [31]. Although this previous research excluded basal and squamous skin cancers, this difference suggests that cancer patients included in the analyses may not be representative of cancer patients from the general population. Besides, there is consistent scientific literature showing that cancer survivors are particularly vulnerable to chronic pain [10,11,12,13,14,15,16,17]. For example, a study including 199 individuals with a history of cancer from the USA revealed that 19.5% of them suffered from chronic pain [10]. It was also observed in another sample of 4526 cancer survivors from that same country that the prevalence of chronic pain was 34.6% [13]. There are substantial differences between these previous works and the present study that may explain, at least partially, the discrepancies in their respective findings. First, prior bodies of research analyzed all types of pain; whereas, this analysis focused on CLBP. Second, the previous studies were cross-sectional and did not include individuals without cancer. In contrast, the present research was of a longitudinal nature and included patients with and without a history of cancer. Regarding the Kaplan–Meier analyses, it is interesting to note that the incidence of CLBP was relatively similar between cancer and noncancer groups within the first three years of follow-up and only increased at a higher rate in people with cancer compared with their counterparts without cancer after the third year. This trend suggests that the factors underlying the cancer–CLBP relationship likely have long-term and prolonged effects. Finally, the fact that more than 15% of patients with and without cancer were diagnosed with CLBP during the 10 years of follow-up warrants critical discussion, as this figure is higher than figures previously reported in the literature. Although the analyses included individuals without any back pain diagnosis in the year preceding the index date, it is possible that back pain might have occurred earlier in the medical history, and individuals with a history of back pain are at particular risk of future episodes of low back pain. When people with back pain diagnosed more than 12 months prior to the index date were excluded from the analyses, the 10-year incidence of CLBP was lower than 10%, corroborating previous findings also obtained in general practices in Germany [21]. The relatively high incidence of CLBP may also be related to the operating definition (i.e., two low back pain diagnoses with more than three months between them), and multiple acute low back pain episodes may have been misclassified as CLBP.

There are several hypotheses that could explain the unexpected relationship between cancer and CLBP. First, physical activity levels may be higher in people with a history of cancer than in the general population. For example, a cross-sectional analysis of 115,257 adults from the Netherlands showed that cancer survivors displayed higher physical activity levels than their counterparts without cancer [32], with regular physical activity being identified as a protective factor against the occurrence of low back pain [20]. Second, it was observed, in a survey conducted in the USA (N = 352 participants), that 46% of smokers stopped smoking following the diagnosis of cancer [33]. At the same time, recent longitudinal evidence from the UK Biobank identified a dose–response relationship between smoking and incident back pain [34]. Third, some data suggest that people with cancer tend to have higher consultation rates than people without cancer. An analysis of data collected in primary care in the United Kingdom discovered that individuals with a history of breast or colorectal cancer had one more consultation per year than individuals without cancer within the five years following the initial cancer diagnosis, while the number of consultations was increased by three for adults with prostate cancer [35]. These results were corroborated in Norway, where some research showed an increased rate of primary care consultations in cancer survivors compared with their counterparts without cancer, with two-thirds of these consultations being unrelated to the history of cancer [36]. In this context, acute low back pain may be diagnosed earlier in people with than in those without cancer, and measures aiming at the prevention of the chronicization of the pain could be implemented more efficiently. Fourth, it may be possible that the diagnosis of cancer has led to occupational changes, with individuals with physically demanding work transiting to a more sedentary occupation. Given that carrying heavy loads at work and difficult working positions are risk factors for CLBP [37], such changes could explain the observed relationship. Fifth, the prescription of analgesics (e.g., paracetamol, opioids, and nonsteroidal anti-inflammatory drugs) is frequent in people with cancer [38], and these treatments may have impacted the incidence of CLBP in this population.

That being said, the present study also identified a positive and significant relationship between cancer and incident CLBP in patients aged >80 years. The positive cancer–CLBP association in older adults could be mediated by sarcopenia. As a matter of fact, a study of 13,761 patients with cancer from China revealed that the prevalence of sarcopenia was around 33% and that increased age was the strongest risk factor for sarcopenia [39]. Meanwhile, preliminary data point toward a positive relationship between sarcopenia and low back pain [40]. This relationship may be stronger in older adults than in young and middle-aged adults, as multimorbidity, functional difficulties, and disability are common in the aged population and may potentiate the deleterious effects of sarcopenia on the occurrence of musculoskeletal pain. Moreover, the association between cancer and incident CLBP was not statistically significant for several cancers (i.e., cancer of the bronchus and lung, cancer of female genital organs, malignant neoplasms of ill-defined, other secondary, and unspecified sites). These cancers are considered relatively aggressive and associated with low survival [41,42], and the mediating factors mentioned above may not apply to these cancers. Finally, the analyses stratified by the presence of metastasis revealed that there was no statistically significant relationship between cancer with metastasis and incident CLBP, suggesting that metastasis may play a substantial role in the occurrence of CLBP in cancer survivors.

### 4.3. Clinical Implications and Directions for Future Research

Pending future confirming studies, this body of research is reassuring, and people with cancer are not at an increased risk for CLBP compared with those without cancer. Nonetheless, based on this study, the incidence of CLBP was high, regardless of the presence of cancer. Given that cancer survivors may already suffer from chronic pain at other anatomical sites, it is essential to improve the prevention of CLBP in this population. Primary care consultations should include education about low back pain, while regular exercise needs to be promoted [43]. In terms of future research, future studies should corroborate or invalidate the present findings in other settings in Germany and other countries and regions of the world.

### 4.4. Strengths and Limitations

The strengths of the study are the large sample size, the duration of the follow-up, and the use of ICD-10 data. Nonetheless, the study results should be interpreted in the light of several limitations. First, low back pain may have been diagnosed in other settings (e.g., rheumatology practices), and this information would not be documented in the Disease Analyzer database. Second, pain related to low back metastasis (i.e., specific low back pain) may have been misclassified as CLBP in the cancer group. Third, there were no data on mortality; although, mortality may have differed between the two groups and is likely a competing risk for incident CLBP, potentially introducing biases in the statistical analyses. Fourth, there was a lack of information on health behaviors (e.g., physical activity and smoking status), quality of life, and functional parameters (e.g., trunk strength and walking speed), and it was, therefore, not possible to adjust the Cox regression analyses for these factors. Some of these variables (e.g., physical activity) may have played a mediating role (e.g., the diagnosis of cancer may have led to an increase in physical activity levels, and physical activity may have been associated with a decrease in the incidence of CLBP). However, these mediating effects could not be investigated. Fifth, data on cancer treatments (i.e., chemotherapy, radiotherapy, and surgery), their efficacy, and their potential complications were insufficiently documented in the database, and it was not possible to investigate the potential impact of these treatments on CLBP. Meanwhile, the prevalence of metastasis at diagnosis or within the first six months was lower than 5%, a figure lower than the one reported in the scientific literature [44], indicating that metastasis may have been underdiagnosed in general practices. Sixth, it is possible that some benign tumors have been misclassified as malignant tumors. Seventh, the study is of a retrospective nature, and the use of prospective data may have led to different conclusions.

### 4.5. Conclusions

In this retrospective cohort exploratory study, including around 407,000 individuals from almost 1300 general practices in Germany, cancer was not statistically associated with an increase in incident CLBP. Despite this finding, the incidence of CLBP was high in the decade following the diagnosis of cancer, underlying the importance of the better prevention of low back pain in cancer survivors, a population at particular risk for multimorbidity. Finally, further research is warranted to corroborate these results before formulating any definite conclusion.

## Figures and Tables

**Table 1:** Characteristics of participants with and without cancer after 1:1 matching.

Variable	People with Cancer (*n* = 203,657)	People Without Cancer (*n* = 203,657)	Standardized Mean Difference ^a^
Age (in years)			
Mean (standard deviation)	66.2 (14.6)	66.0 (13.8)	−0.014
≤50	27,501 (13.5)	29,129 (14.3)	
51–60	37,039 (18.2)	36,890 (18.1)	
61–70	52,377 (25.7)	48,739 (23.9)	
71–80	58,582 (28.8)	55,229 (27.1)	
>80	28,158 (13.8)	33,670 (16.5)	
Sex			
Female	104,509 (51.3)	105,431 (51.8)	−0.005
Male	99,148 (48.7)	98,226 (48.2)	
Consultations per year during the follow-up, mean (standard deviation)	8.7 (3.8)	8.8 (3.8)	−0.026
Index year			
2005–2007	13,463 (6.6)	14,437 (7.1)	−0.047
2008–2010	22,718 (11.2)	20,232 (9.9)	
2011–2013	33,299 (16.4)	30,136 (14.8)	
2014–2016	41,611 (20.4)	40,529 (19.9)	
2017–2019	49,816 (24.5)	52,109 (25.6)	
2020–2022	42,750 (21.0)	46,214 (22.7)	
Chronic conditions			
Chronic ischemic heart disease	30,330 (14.9)	29,863 (14.7)	−0.002
Chronic obstructive pulmonary disease	19,206 (9.4)	18,921 (9.3)	−0.001
Depression	32,252 (15.8)	32,422 (15.9)	0.001
Diabetes mellitus	43,060 (21.1)	43,934 (21.6)	0.004
Disorders of lipoprotein metabolism and other lipidemias	58,974 (29.0)	59,035 (29.0)	0.000
Obesity	17,842 (8.8)	17,537 (8.6)	−0.001
Sleep disorders	22,946 (11.3)	22,493 (11.0)	−0.002
Somatoform disorders	19,376 (9.5)	18,943 (9.3)	−0.002

Data are absolute numbers (percentages) unless otherwise specified. People with cancer were matched (1:1) to those without cancer using a propensity score based on age, sex, the mean number of consultations per year during the follow-up, index year, and several conditions (i.e., chronic ischemic heart disease, chronic obstructive pulmonary disease, depression, diabetes mellitus, disorders of lipoprotein metabolism and other lipidemias, obesity, sleep disorders, and somatoform disorders). ^a^ The standardized mean difference is frequently used to analyze the distribution of covariates after propensity score matching, with differences higher than 0.1 corresponding to some imbalance. Regarding age, the standardized mean difference was computed for the continuous variable only.

**Table 2:** Frequent conditions not included in the propensity score diagnosed in individuals with and without cancer.

Variable	People with Cancer (*n* = 203,657)	People Without Cancer (*n* = 203,657)	*p*-Value ^a^
Atrial fibrillation and flutter	16,767 (8.2)	17,128 (8.4)	0.041
Disorders of the thyroid gland	44,290 (21.7)	43,434 (21.3)	0.001
Essential hypertension	96,458 (47.4)	99,908 (49.1)	<0.001
Gastritis and duodenitis	31,304 (15.4)	31,935 (15.7)	0.006
Gastro-esophageal reflux disease	26,824 (13.2)	25,736 (12.6)	<0.001
Heart failure	16,103 (7.9)	16,317 (8.0)	0.215
Osteoarthritis of the knee	22,464 (11.0)	21,398 (10.5)	<0.001
Varicose veins of the lower extremities	17,649 (8.7)	17,062 (8.4)	0.001

Data are absolute numbers (percentages) unless otherwise specified. ^a^ *p*-values were obtained using the McNemar test.

**Table 3:** Most frequent cancer diagnoses in the cancer cohort.

Variable	Patients with Cancer (*n* = 203,657)
Breast cancer	29,969 (14.7)
Cancer of bronchus and lung	8360 (4.1)
Cancer of esophagus or stomach	4291 (2.1)
Cancer of female genital organs	9114 (4.5)
Colorectal cancer	17,536 (8.6)
Malignant neoplasms of ill-defined, other secondary, and unspecified sites	14,544 (7.1)
Malignant neoplasms of lymphoid, hematopoietic, and related tissue	15,411 (7.6)
Prostate cancer	23,082 (11.3)
Skin cancer	45,409 (22.3)
Urinary tract cancer	13,882 (6.8)
Cancer of other sites	22,059 (10.8)

Data are absolute numbers (percentages).

**Table 4:** Association between cancer and incident chronic low back pain in people followed for up to 10 years in general practices in Germany.

Population	Hazard Ratio (95% Confidence Interval)	*p*-Value
Overall population	0.87 (0.86–0.89)	<0.001
By age (in years)		
≤50	0.87 (0.83–0.92)	<0.001
51–60	0.78 (0.75–0.82)	<0.001
61–70	0.83 (0.80–0.87)	<0.001
71–80	0.98 (0.94–1.02)	0.313
>80	1.12 (1.04–1.19)	0.001
By sex		
Female	0.88 (0.86–0.91)	<0.001
Male	0.86 (0.84–0.89)	<0.001
By cancer diagnosis		
Breast cancer	0.76 (0.72–0.80)	<0.001
Cancer of bronchus and lung	1.01 (0.92–1.12)	0.799
Cancer of esophagus or stomach	0.86 (0.75–0.99)	0.039
Cancer of female genital organs	1.07 (0.97–1.18)	0.165
Colorectal cancer	0.83 (0.77–0.89)	<0.001
Malignant neoplasms of ill-defined, other secondary, and unspecified sites	0.96 (0.89–1.04)	0.329
Malignant neoplasms of lymphoid, hematopoietic, and related tissue	0.88 (0.81–0.95)	<0.001
Prostate cancer	0.80 (0.75–0.86)	<0.001
Skin cancer	0.94 (0.90–0.98)	0.004
Urinary tract cancer	0.90 (0.83–0.97)	0.008
Cancer of other sites	0.86 (0.81–0.91)	<0.001
By metastasis		
Absence of metastasis	0.88 (0.86–0.90)	<0.001
Presence of metastasis	1.04 (0.93–1.15)	0.510

Hazard ratios and 95% confidence intervals were obtained using Cox regression models with cancer as the independent variable, chronic low back pain as the dependent variable, and the conditions listed in Table 2 as control variables. Metastasis was assessed at the index date and within the first six months of follow-up.

## Data Availability

The data and the code used for this study are available from the corresponding author upon reasonable request.

## Supplementary Materials

The following supporting information can be downloaded at: https://www.mdpi.com/article/10.3390/jcm13226969/s1, Table S1: STROBE Statement—Checklist of items that should be included in reports of cohort studies.
