# Supplementary material for: Cancer and the 10-Year Incidence of Chronic Low Back Pain in 407,314 Adults Followed in General Practices in Germany
Source: J Clin Med. 2024 Nov 19;13(22):6969. doi: 10.3390/jcm13226969 (PMC11595432; doi:10.3390/jcm13226969)
Supplement: Supplementary file 1 [file jcm-13-06969-s001.zip › jcm-3235844-supplementary.pdf]

Supplementary Table S1. STROBE Statement—Checklist of items that should be included in reports of cohort studies

	Item No	Recommendation	Page No
**Title and abstract**	1	(*a*) Indicate the study's design with a commonly used term in the title or the abstract	Abstract; Objective
		(*b*) Provide in the abstract an informative and balanced summary of what was done and what was found	Abstract; Methods and Results
**Introduction**			
Background/rationale	2	Explain the scientific background and rationale for the investigation being reported	Introduction; first and second paragraph
Objectives	3	State specific objectives, including any prespecified hypotheses	Introduction; third paragraph
**Methods**			
Study design	4	Present key elements of study design early in the paper	Materials and methods; Database and Study population
Setting	5	Describe the setting, locations, and relevant dates, including periods of recruitment, exposure, follow-up, and data collection	Methods; Study population
Participants	6	(*a*) Give the eligibility criteria, and the sources and methods of selection of participants. Describe methods of follow-up	Methods; Study population and Figure 1
		(*b*) For matched studies, give matching criteria and number of exposed and unexposed	Methods; Study population and Figure 1
Variables	7	Clearly define all outcomes, exposures, predictors, potential confounders, and effect modifiers. Give diagnostic criteria, if applicable	Methods; Study outcome and Study covariates
Data sources/ measurement	8*	For each variable of interest, give sources of data and details of methods of assessment (measurement). Describe comparability of assessment methods if there is more than one group	Methods; Database, Study outcome, and Study covariates

Bias	9	Describe any efforts to address potential sources of bias	Methods; Statistical analyses
Study size	10	Explain how the study size was arrived at	Methods; Study population and Figure 1
Quantitative variables	11	Explain how quantitative variables were handled in the analyses. If applicable, describe which groupings were chosen and why	Methods; Statistical analyses
Statistical methods	12	(*a*) Describe all statistical methods, including those used to control for confounding	Methods; Statistical analyses
		(*b*) Describe any methods used to examine subgroups and interactions	Methods; Statistical analyses
		(*c*) Explain how missing data were addressed	Methods; Statistical analyses
		(*d*) If applicable, explain how loss to follow-up was addressed	Methods; Statistical analyses and Figure 2
		(*e*) Describe any sensitivity analyses	Methods; Statistical analyses
**Results**			
Participants	13*	(a) Report numbers of individuals at each stage of study—eg numbers potentially eligible, examined for eligibility, confirmed eligible, included in the study, completing follow-up, and analysed	Figure 1
		(b) Give reasons for non-participation at each stage	Figure 1
		(c) Consider use of a flow diagram	Figure 1
Descriptive data	14*	(a) Give characteristics of study participants (eg demographic, clinical, social) and information on exposures and potential confounders	Results; Characteristics of the population and Tables 1-3
		(b) Indicate number of participants with missing data for each variable of interest	Not applicable
		(c) Summarise follow-up time (eg, average and total amount)	Results; Characteristics of the population
Outcome data	15*	Report numbers of outcome events or summary measures over time	Figure 2

Main results	16	(*a*) Give unadjusted estimates and, if applicable, confounder-adjusted estimates and their precision (eg, 95% confidence interval). Make clear which confounders were adjusted for and why they were included	Figure 2 and Table 4
		(*b*) Report category boundaries when continuous variables were categorized	Table 4
		(*c*) If relevant, consider translating estimates of relative risk into absolute risk for a meaningful time period	Not applicable
Other analyses	17	Report other analyses done—eg analyses of subgroups and interactions, and sensitivity analyses	Table 4
**Discussion**			
Key results	18	Summarise key results with reference to study objectives	Discussion; Main findings
Limitations	19	Discuss limitations of the study, taking into account sources of potential bias or imprecision. Discuss both direction and magnitude of any potential bias	Discussion; Strengths and limitations
Interpretation	20	Give a cautious overall interpretation of results considering objectives, limitations, multiplicity of analyses, results from similar studies, and other relevant evidence	Discussion; Interpretation of the findings
Generalisability	21	Discuss the generalisability (external validity) of the study results	Discussion; Interpretation of the findings, Clinical implications and directions for future research, and Conclusions
**Other information**			
Funding	22	Give the source of funding and the role of the funders for the present study and, if applicable, for the original study on which the present article is based	Funding

*Give information separately for exposed and unexposed groups.

Note: An Explanation and Elaboration article discusses each checklist item and gives methodological background and published examples of transparent reporting. The STROBE checklist is best used in conjunction with this article (freely available on the Web sites of PLoS Medicine at http://www.plosmedicine.org/, Annals of Internal Medicine at http://www.annals.org/, and Epidemiology at http://www.epidem.com/). Information on the STROBE Initiative is available at http://www.strobe-statement.org.
